# Bridging, Mapping, and Addressing Research Gaps in Health Sciences: The Naqvi-Gabr Research Gap Framework

**DOI:** 10.7759/cureus.55827

**Published:** 2024-03-08

**Authors:** Waqar M Naqvi, Mamdouh Gabr, Sakshi P Arora, Gaurav V Mishra, Aishwarya A Pashine, Zahiruddin Quazi Syed

**Affiliations:** 1 Faculty of Interdisciplinary Sciences, Jawaharlal Nehru Medical College, Datta Meghe Institute of Higher Education and Research, Wardha, IND; 2 Department of Physiotherapy, College of Health Sciences, Gulf Medical University, Ajman, ARE; 3 Department of Radiodiagnosis, Jawaharlal Nehru Medical College, Datta Meghe Institute of Higher Education and Research, Wardha, IND; 4 Department of Cardiorespiratory Physiotherapy, Career College Bhopal, Bhopal, IND; 5 Department of Community Medicine, Jawaharlal Nehru Medical College, Datta Meghe Institute of Higher Education and Research, Wardha, IND

**Keywords:** educational research, framework, naqvi-gabr research gap, health sciences, research gaps

## Abstract

Innovations pertaining to the ever-evolving needs of the medical and healthcare sciences remain constant. This creates a gap between the rationalized needs of the study and the proposed research question. However, classifying, identifying, and addressing these research gaps require a systematic and precise structured map. Using the Medical Subject Heading (MeSH) terms “Research Gaps” AND “Healthcare” AND “Framework” in MEDLINE, Scopus, and CINAHL databases with the filters yielded no relevant literature. Therefore, this review aims to fill this practical and clinical knowledge gap by developing the Naqvi-Gabr Research Gap Framework through critical synthesis based on extensive research on medical and healthcare research gaps. Fourteen research gaps are distributed for allocation as per the healthcare delivery system approach: developing new treatments or prevention strategies, improving diagnostic tools and techniques, addressing health disparities, and improving access to healthcare services. This structured framework determines the strategic mapping of research gaps corresponding to the nature of the research. The identification and classification of the appropriate research gap led to precise and concise conclusions corresponding to the research process proposed in this study. Hence, the Naqvi-Gabr Research Gap Framework is a valuable tool for determining the potential application of gaps by researchers, policymakers, and other stakeholders with a productive address.

## Introduction and background

In the healthcare sciences, research gaps fill various dimensions of healthcare systems, ranging from basic scientific research [1] to clinical trials [2] in the healthcare delivery hierarchy [3]. Identifying a particular research gap is essential for guiding future research [4] and improving healthcare outcomes specific to the targeted population [5], interventions [6], and outcomes [7] dealing with various challenges and opportunities [8].

There are several methods for identifying health research gaps, establishing research needs, and determining research priorities, including scoping reviews, systematic reviews, Delphi surveys, expert panels, and stakeholder consultations [9,10]; however, there remains a lacuna of structured universal reference rationalizing the identity and address of the research gaps in medical sciences. Even though, the literature presents knowledge translation closing the gap between evidence and practice [11], a concise and precise framework for classifying, identifying, and mapping these gaps in healthcare research is required. This builds a need for a universal reference to systematically identify research gaps in the potential need to classify the limitations of the current evidence [8,12]. Hence, this study aimed to determine the different facets of healthcare to derive a comprehensive, explicit, and viable Naqvi-Gabr Research Gap framework to map and bridge the open ends of a hypothesis improving the quality and effectiveness of healthcare research and practice.

## Review

Materials and methods

A search was carried out with the Medical Subject Heading (MeSH) terms “Research Gaps” AND “Healthcare” OR “Health” AND “Framework” in MEDLINE, Scopus and CINAHL databases with the filters applied showing results free full-text review articles published in last five years (2018 to 2023) on humans in the English language with the filters yielded no relevant literature. Therefore, this review aims to fill this practical and clinical knowledge gap by developing the Naqvi-Gabr Research Gap Framework through critical synthesis-based extensive research on medical and healthcare research gaps.

Results

**Figure 1:**
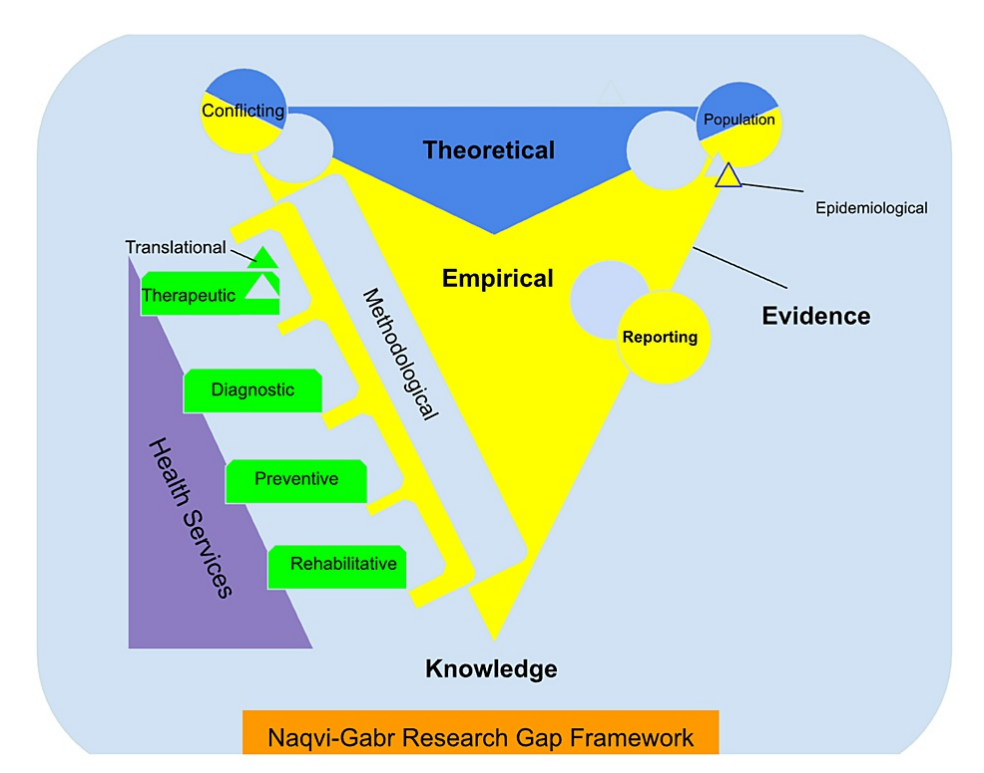
A simplified map for allocating the 14 identified and proposed research gaps in healthcare sciences as the Naqvi-Gabr Research Gap Framework.

The framework identifies 14 research gaps in healthcare sciences following two elements of classification: the characterization of the gaps and the identification and classification of the reason for allocating the gap [8]. It includes knowledge, evidence, reporting, epidemiological, empirical, prevention, diagnosis, methodological, therapeutic, translation, rehabilitation, health services, theoretical, and conflict gaps [9,13] as shown in Figure 1.

Discussions

The types of research gaps explained in the framework are as follows:

*1.* *Knowledge Gap*

The knowledge gap in health research refers to unstructured information without evidence, structured evidence-based information, or both. This gap is pertinent when the existing literature is insufficient to answer a particular research question [14]. The body of knowledge is represented in the framework by a blue-sky area, where the triangle represents evidence. Evidence indicates proven knowledge, both theoretically and empirically. Knowledge is differentiated as evidence-based if it has theoretical and experimental data; otherwise, an evidence gap is identified.

*2.* *Evidence Gap*

The evidence gap refers to areas where there is a lack of robust or high-quality scientific evidence to support or guide clinical decision-making or healthcare interventions [15]. This may involve a scarcity of randomized controlled trials (RCTs), systematic reviews, or meta-analyses of specific interventions or outcomes [16]. Effectiveness of new treatments stating if new medications, therapies, or surgical procedures are introduced, there may be limited evidence on their safety, efficacy, and long-term outcomes.

*3.* *Reporting Gap*

Effective research is characterized by validity and reliability [17] but following a lack of awareness, the literature lacks detailed specifications of the experimental design, sample size, the component of the drugs, or the treatment protocol followed, limiting the repetition of the experiments [18]. This may be because of financial conflicts or trading concerns, restricting the utility of the right to report, and hence, can be considered a reporting gap. Identifying the reporting gap would motivate researchers to try the same experiments and report missing segments in the body of evidence. Reporting gaps occur when evidence is not reported in sufficient detail to allow for replication or meta-analysis. From another point of view, experiments can be assessed for test-retest reliability, and by default, it is considered an empirical gap [19], where the accuracy of true experiments should support the main theory.

*4.* *Epidemiological Gap*

An epidemiological gap arises when there is insufficient information regarding the distribution and determinants of a disease or health condition in a particular population [20]. Epidemiological studies play a critical role in the understanding of the transmission dynamics of infectious diseases. However, there may be gaps in knowledge regarding the modes of transmission, reservoirs, and factors influencing disease spread, particularly for emerging or re-emerging infections. The population gap in health sciences refers to the under representation or insufficient inclusion of specific populations in studies and clinical trials [21], leading to a limited understanding of health outcomes and interventions on the basis of criteria including gender-based gaps referring to the biomedical research traditionally focused on males, leading to a lack of understanding of women's health needs and treatment responses [22]; racial and ethnic gaps referring to the disparities in health outcomes and disease prevalence existing due to limited representation and data from diverse racial and ethnic groups in research studies [23]; age-related gaps imposing under representation of older adults in clinical trials limiting knowledge about effective treatments and interventions for this population; socioeconomic gaps covering the lower socioeconomic status can hinder research participation, resulting in inadequate representation and limited understanding of health outcomes among disadvantaged populations; and geographic gaps involving research conducted in specific regions may not accurately represent populations from rural or remote areas, impacting the generalizability of findings and interventions.

5. Empirical Gap

The other part of the evidence is empirical or experimental [19]. The postulated theories require experimental support to withstand or outstand the hypotheses identified. Thus, this creates an empirical gap. An empirical study is a procedure undertaken to support or refute a hypothesis or determine the efficacy of something previously overlooked. Experiments provide insight into cause-and-effect by demonstrating the outcome when any associated variable is altered. It aims to identify areas where there is a lack of data, or where the available data are inconsistent or incomplete. This gap represents a sensitive research area to expand knowledge [15], address unanswered questions, and provide a solid evidence base for healthcare practice. Identifying empirical gaps is an essential step in the research process as it helps researchers and policymakers determine the focus of their research question and allocate resources effectively. By addressing this gap, researchers can contribute to advancements in healthcare, improve patient outcomes, and synthesize evidence-based decision-making for practitioners. There may be gaps in the empirical evidence [24] regarding the effectiveness of preventive interventions, such as vaccinations, screening programs, or lifestyle modifications, in reducing the incidence or progression of diseases.

6. Prevention Gap

The prevalence of chronic diseases places a significant burden on healthcare worldwide following a lack of effective prevention strategies, implementation, or research for a specific health condition in specific populations or subgroups, particularly in low-resource settings or marginalized communities, which is referred to as the prevention gap. As per the research gap framework, it is considered a part of the methodology, but it has an impact on health services, which require adequate funds and health authorities to ensure the effective delivery of the disease prevention program to the population. The National Institutes of Health (NIH) Office of Disease Prevention (ODP) [25], National Academies [26], US Preventive Services Task Force (USPSTF) [27], and JAMA Network [28] identify and report this gap.

7. Diagnosis Gap

In remote healthcare settings, access to advanced diagnostic tools, specialized tests, or experienced healthcare professionals is limited. This results in a diagnostic gap, as patients may not receive timely and accurate diagnoses owing to resource constraints [29]. The diagnosis gap refers to the difference between the provisional diagnosis and the final diagnosis of a disease or condition. This gap can occur due to a lack of knowledge or skill of the treating doctors, noncompliance with standard diagnostic criteria, or statutory lapses by hospital authorities in the documentation of medical records [30]. The diagnostic and diagnosis gaps are significant issues in global health, particularly in low- and middle-income countries where the diagnostic capacity gap in primary care is enormous. This cannot be completely accessed with the existing diagnostic facilities, many of which are costly, require laboratory infrastructure, and are highly trained operators. The diagnostic gap is also a significant issue in the diagnosis of tuberculosis, where gaps can occur owing to the use of suboptimal diagnostic tests, lack of specialty services, knowledge and behavior of healthcare professionals, attitude and behavior of patients, and poor adherence to programmatic diagnosis algorithms [31]. The diagnostic gap is also an issue in hypertension, in which a significant percentage of the population has not been diagnosed [18]. Finally, a diagnostic gap can occur in rare diseases, where individuals may experience gaps between symptoms, diagnosis, and treatment when in need of care. The diagnostic gap originates as a part of the research methodology, and in turn, it depends on health services such as funds, caregiver experiences, and health authorities.

8. Methodological Gap

While conducting an experimental study, a group of participating subjects served as a representative of the entire population from which they were recruited, overlaying radical results irrespective of effects [32], and creating a gap, which is called the methodological gap. This occurs when there is a lacuna in the research methodology or design. If a study on the effectiveness of a drug includes only a specific age group or excludes individuals with comorbidities, the findings may not be generalizable to the broader population, leading to a methodological gap. This methodological gap may highlight faults in the choice of research design or the type of statistical analysis that enhances research bias and affects the reliability of the research results. Therefore, researchers have attempted to repeat the same experimental study using different statistical analyses or study designs to obtain more accurate results, focusing on the validity and reliability of measurement tools [17]. Identifying methodological gaps is important because it can lead to more reliable research outcomes. Researchers should identify methodological gaps at each stage of the research process to ensure that the research is unbiased until completion.

9. Therapeutic Gap

For instance, in rare disorders, a therapeutic gap can be identified, and effective treatment is lacking. It is considered a gap of methodological origin [32]; however, the limited access to effective existent treatment due to the cost effect of lack of accessibility, as in developing countries, makes it a health-services-dependent gap. Mental health disorders such as depression, anxiety, and schizophrenia often face therapeutic gaps. Existing treatments may not be effective in all patients, and there is a need for improved interventions with better efficacy and tolerability. Developing innovative therapies [25], exploring new target drugs, and advancing psychotherapeutic approaches are essential for filling this therapeutic gap.

10. Translation Gap

While testing the hypothesis of RCTs [33], the probability of errors was sustained. This creates a gap in the availability of treatment but lacks applicability only within a part of the therapeutic effect called the translational gap. These gaps arise when evidence from RCTs is not effectively translated into clinical practice [9]. For example, if a highly effective intervention is not widely and properly adopted in clinical practice or not conducted effectively, it may lead to misleading results that specify a gap in the translation of knowledge.

11. Rehabilitation Gap

The rehabilitation gap refers to the lack of access to evidence-based rehabilitation along with physical therapy at all stages of care. This gap appears to be caused by patients being allowed (or able to afford) a certain number of appointments [34]. The rehabilitation gap refers to the period after a patient has undergone surgery or treatment but before they have fully recovered, during which they may not have access to physical therapy or may be prohibited by the cost effects, referring to the health service-dependent gap. The rehabilitation gap originated from the methodology of the research but the narrowed research to practice translation restricts the knowledge application.

12. Health Service Gap

Experimental studies require funds, approval from governmental ethical committees, and an eligible healthcare delivery system [9]. Hence, the health services gap can originate from a lack of supporting funds, healthcare delivery, or the powers of healthcare authorities [3]. Health service gaps occur when there is a lack of access to or availability of healthcare services for a particular health condition [6]. Health service gaps may also occur when research on the effectiveness and cost-effectiveness of certain healthcare services is lacking.

13. Theoretical Gap

When the postulated theories fail to address a concept or a phenomenon, it creates a gap of limitations of existing theories or frameworks, termed a theoretical gap. The clinical trials or comparative studies planned based on theories determine the overlooked segments of either the interpretation or explanation of the theory. This approach seeks the revision of the existing concept or the proposal of a novel concept [19,35].

14. Conflict Gap

Conflict Gap occurs when two or more research studies have different findings, results, or conclusions regarding the same research question. So the research question that needs to be answered is which of the conflicted results is true creating the conflict gap to be addressed [36]. On the other hand, a conflict gap might be present between the theories used to describe the same concept or phenomena making it a theoretical conflict gap.

## Conclusions

Identifying and addressing research gaps in the medical and healthcare sciences are crucial for improving healthcare outcomes. This study highlights a structured universal framework inculcating various research gaps and emphasizes the importance of targeted research efforts and evidence-based interventions to bridge these gaps. Collaboration among researchers, clinicians, and policymakers is necessary to prioritize research funding and translate research findings into clinical practice. By addressing these gaps, healthcare services can be advanced, patient outcomes can be improved, and decision-making can be alleviated. Future research should focus on developing strategies to close these gaps and ensure high-quality and effective healthcare research and practices.
